# A multi-template combination algorithm for protein comparative modeling

**DOI:** 10.1186/1472-6807-8-18

**Published:** 2008-03-17

**Authors:** Jianlin Cheng

**Affiliations:** 1 Department of Computer Science, Informatics Institute, University of Missouri, Columbia, MO 65211-2060, USA

## Abstract

**Background:**

Multiple protein templates are commonly used in manual protein structure prediction. However, few automated algorithms of selecting and combining multiple templates are available.

**Results:**

Here we develop an effective multi-template combination algorithm for protein comparative modeling. The algorithm selects templates according to the similarity significance of the alignments between template and target proteins. It combines the whole template-target alignments whose similarity significance score is close to that of the top template-target alignment within a threshold, whereas it only takes alignment fragments from a less similar template-target alignment that align with a sizable uncovered region of the target.

We compare the algorithm with the traditional method of using a single top template on the 45 comparative modeling targets (i.e. easy template-based modeling targets) used in the seventh edition of Critical Assessment of Techniques for Protein Structure Prediction (CASP7). The multi-template combination algorithm improves the GDT-TS scores of predicted models by 6.8% on average. The statistical analysis shows that the improvement is significant (p-value < 10^-4^). Compared with the ideal approach that always uses the best template, the multi-template approach yields only slightly better performance. During the CASP7 experiment, the preliminary implementation of the multi-template combination algorithm (FOLDpro) was ranked second among 67 servers in the category of high-accuracy structure prediction in terms of GDT-TS measure.

**Conclusion:**

We have developed a novel multi-template algorithm to improve protein comparative modeling.

## Background

Protein structure prediction is one of the most important problems in structural bioinformatics [1-3]. Comparative (or homology) modeling is currently the most accurate and practical structure prediction method [4-19].

In general comparative modeling involves four steps [11,20,21]: (1) identify a homologous template protein for a target protein; (2) generate an alignment between the template and the target; (3) create a model based on the alignment and the template structure; (4) evaluate and refine the model. The two key factors determining the quality of comparative modeling are the template structure and the alignment accuracy [22]. Traditionally, automated comparative modeling methods use the top-ranked template and its alignment with the target protein to model its structure. This approach cannot always achieve the best results because it may not be able to select the best template and to generate the optimal alignment [23]. Previous research [6,24-28], particularly the human prediction [23,29-31] in the six edition of Critical Assessment of Techniques of Protein Structure Prediction (CASP6) [32-36], has shown that using multiple templates can often improve the quality of comparative modeling over a single template. Although human experts commonly select multiple templates and combine them manually to predict structure in their practice, multiple-template combination has not been widely used by automated servers until the latest CASP7 experiment, 2006. In CASP7, several servers including FOLDpro and 3Dpro [37,38], HHSearch [39], 3D-JIGSAW-POPULUS [13], MetaTasser [30,40,41], Zhang-Server [29], FAMS [42], used multiple templates to improve template-based structure prediction. Some of these methods such as Zhang-Server, FOLDpro, and 3Dpro show the good performance on the comparative modeling targets, particularly on the high-accuracy modeling targets. However, few of the automated multi-template methods are published so far.

A published multiple-template algorithm [29-31] tries to extract distance (or contact) restraints from multiple templates. The consistent distance restraints from multiple templates are used to guide structure modeling. This method is currently coupled with the in-house model assembly tool TASSER [29,31] and cannot be used with the popular, publicly available, comparative model generation tools such as Modeller [8,11,20,43,44], nest [17], SEGMOD-ENCAD [45], SWISS-Model [18,46,47], 3D-JIGSAW [13], and Builder [9]. Most of these tools take as inputs the alignments between templates and a target to generate structure models, instead of directly accepting distance restraints.

Thus, instead of extracting distance restraints from multiple templates as in [29-31], we develop a different, parametric algorithm to select templates and to combine their alignments directly. The multiple alignments between the target and the templates can be directly fed into the widely used, standard comparative modeling tools such as Modeller [8] with the built-in multi-template modeling function, to generate models. The preliminary implementation of the method was ranked second in the automated high-accuracy structure prediction during the CASP7 community-wide experiment.

Furthermore, we systematically and rigorously compare the performance of the multiple- and single-template methods on the 45 comparative modeling targets of the CASP7 experiment. On average the multiple-template combination algorithm improves the GDT-TS score [48] of the predicted models by 6.8% over the single template approach. The pairwise statistical analysis shows that the improvement is significant. Thus, our experiment strictly demonstrates that the combination of multiple templates and their alignments can *significantly *improve comparative modeling over the single top template approach. Moreover, we compare the multi-template method against the ideal approach, which always uses the best, possible template in the Protein Data Bank [49]. The analysis shows that the multi-template combination algorithm can even achieve slightly better performance than the ideal approach on the 27 CASP7 comparative modeling targets. However, the improvement is not statistically significant.

## Results and Discussions

**Figure 1:**
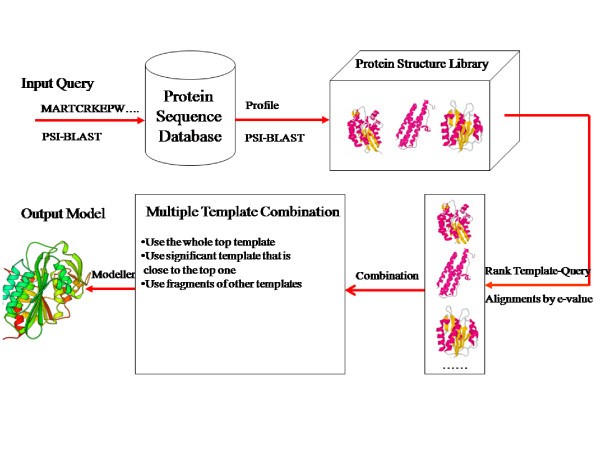
An automated multi-template comparative modeling pipeline.

We develop a pipeline for multi-template protein comparative modeling as shown in Figure 1. Given an input target (or query) protein, the pipeline uses PSI-BLAST [50] to search homologous structure templates. The target-template alignments ranked by PSI-BLAST e-values are combined with respect to the target protein. Briefly speaking, the algorithm always uses the most significant template-target alignment. The other significant alignments relative to the most significant one are also automatically included. The less significant template-target alignments are chosen only if they can align with a continuous region of the target that is not covered by the previously selected template-target alignments. And only the alignment fragments that align with the uncovered regions are used. The combined alignments and template structures are fed into Modeller [8] to generate structure models for the target protein. The details of the algorithm are described in the Methods section.

Our multi-template combination method was first blindly tested in the CASP7 experiment, 2006. Since then we systematically evaluate the algorithm on the CASP7 comparative modeling targets. A target is classified as a comparative modeling target if a structure template covering all the domains of the target can be found by PSI-BLAST search as in [51]. Here, we firstly compare the multi-template combination algorithm with a single-top-template algorithm on all the comparative modeling targets. Secondly, we compare two approaches on the high-accuracy comparative modeling targets. Thirdly, we compare the multi-template combination algorithm against the ideal approach that always uses the best template. Fourthly, we compare the multi-template combination method against the other automated methods and the human predictors in the CASP7 experiment. Finally, we discuss why the multi-template approach improves model quality.

The models of the multi-template combination algorithm were generated during the CASP7 period when the structures of the targets were not known. For the comparision we use Modeller [8] to generate 3D structure models for the single top template and the best template respectively, based on the alignments generated by PSI-BLAST during the CASP7 experiment. We use LGA [48], a sequence-dependent structure alignment tool, to evaluate the models against the experimental structures to get GDT-TS scores.

### Comparison with the Single Top Template Approach on the CASP7 Comparative Modeling Targets

We compare the multi-template combination algorithm with the single-template algorithm on the 45 CASP7 comparative modeling targets, for which PSI-BLAST can identify at least two significant templates. The other two comparative modeling targets (T0326 and T0328) that have only one template found by PSI-BLAST are excluded. The single-template algorithm always uses the most significant template with the lowest e-value of PSI-BLAST.

**Figure 2:**
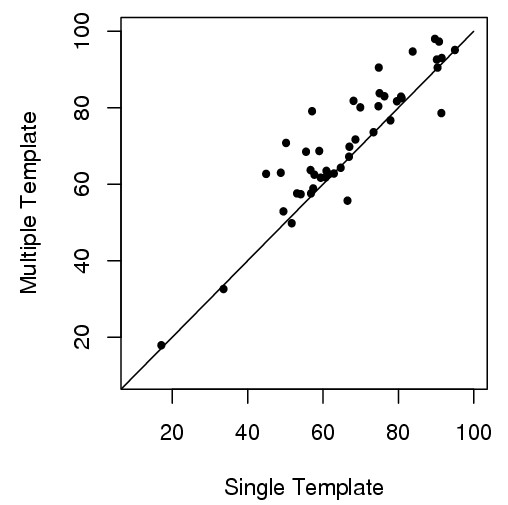
**GDT-TS scores of 45 comparative modeling targets (multi-template versus single-template)**. For 38 out of 45 targets, the multi-template approach yields higher GDT-TS scores than the single-template approach. The dots above the line represent the targets where the multi-template method yields higher scores, on the line where two methods yields the same scores, and below the line where the single-template method yields higher scores.

Table 1 shows the GDT-TS scores of 45 targets using the single- and multi-template methods, respectively. The number of templates used by the multi-template combination algorithm ranges from 2 to 39. The average number of templates used is 12.4. According to the results, the multi-template combination improves GDT-TS score for the majority of cases (38 out of 45 targets) as shown in Figure 2, consistent with the previous human prediction experiment [23,31]. The average score of using multiple templates is 71.15 versus 66.59 of using the single most significant template. The average improvement of GDT-TS score is 6.8% (raw score increase = 4.56).

**Table 1:** The results of the multiple- and single-template methods on the 45 comparative modeling targets of CASP7.

Target Id	Temp Num	Multi	Single	Multi – Single
T0288	27	83.8	75.0	8.8
T0290	14	97.3	90.8	6.5
T0291	6	78.6	91.4	-12.8
T0292	37	69.8	67.0	2.8
T0293	15	32.6	33.6	-1.0
T0294	18	81.8	68.1	13.7
T0295	2	83.0	76.3	6.7
T0297	8	62.8	62.9	-0.1
T0298	39	70.8	50.2	20.6
T0302	26	80.1	69.9	10.2
T0303	20	68.7	59.0	9.7
T0305	5	93.0	91.5	1.5
T0308	19	90.5	74.8	15.7
T0310	6	55.7	66.5	-10.8
T0313	12	80.4	74.7	5.7
T0315	4	94.7	83.8	10.9
T0316	9	17.9	17.1	0.8
T0317	8	81.7	79.6	2.1
T0318	4	58.9	57.4	1.5
T0322	22	68.5	55.5	13.0
T0323	20	57.6	53.1	4.5
T0324	18	79.1	57.1	22.0
T0329	22	63.0	48.8	14.2
T0330	23	62.7	44.9	17.8
T0332	2	82.9	80.7	2.2
T0337	4	52.9	49.5	3.4
T0338	17	49.8	51.7	-1.9
T0339	7	76.7	77.9	-1.2
T0340	4	90.5	90.4	0.1
T0341	6	67.2	66.9	0.3
T0345	3	95.1	95.0	0.1
T0346	8	98.0	89.7	8.3
T0359	15	82.5	80.9	1.6
T0362	12	73.6	73.4	0.2
T0364	23	71.7	68.6	3.1
T0366	3	92.6	90.2	2.4
T0371	6	61.7	59.4	2.3
T0373	12	62.6	61.5	1.1
T0374	5	62.5	57.7	4.8
T0375	6	57.4	54.1	3.3
T0376	14	64.3	64.7	-0.4
T0379	6	63.5	60.9	2.6
T0380	9	63.7	56.7	7.0
T0381	2	57.6	56.8	0.8
T0384	11	61.8	60.7	1.1

Average	12.42	71.15	66.59	4.56

Column 1 lists the CASP7 protein target IDs. Column 2 lists the number of templates used by the multi-template combination algorithm. Column 3 lists the GDT-TS scores of the multi-template algorithm for the targets. Column 4 lists the GDT-TS scores of using the most significant single template with the lowest e-value. Column 5 lists the GDT-TS score difference between the multiple- and single-template approaches.

We conduct a paired t-test (t-value = 4.39, the degree of freedom = 44) on the GDT-TS scores of 45 targets. The p-value of getting an average score difference >= 4.56 is 3.5 × 10^-5 ^under the hypothesis that the difference is 0. So combining multiple templates and their alignments significantly improves the quality of comparative modeling over the single-template approach.

### Comparison with the Single Top Template Approach on the CASP7 High-Accuracy Modeling Targets

A special category of template-based modeling (i.e. high-accuracy modeling), where models have GDT-TS scores > 80 compared to experimental structures, is particularly useful for understanding protein function at the atomic level. To emphasize its importance, CASP7 dedicated a category of high-accuracy template-based modeling to evaluate methods on the targets for which there is at least one template with LGA-S score > 80 and at least one method produced a model with GDT-TS > 80. Using this criteria, CASP7 classifies 28 domains from 24 protein targets into the high-accuracy modeling category. Among them T0326 and T0328 have only one template. The templates for T0311 and T0367 cannot be found by PSI-BLAST. The structure of T0334 is not released at the time of writing the paper. So we exclude these five targets and select the remaining 23 high-accuracy domains to compare the multiple-template combination algorithm with the single-template approach.

**Figure 3:**
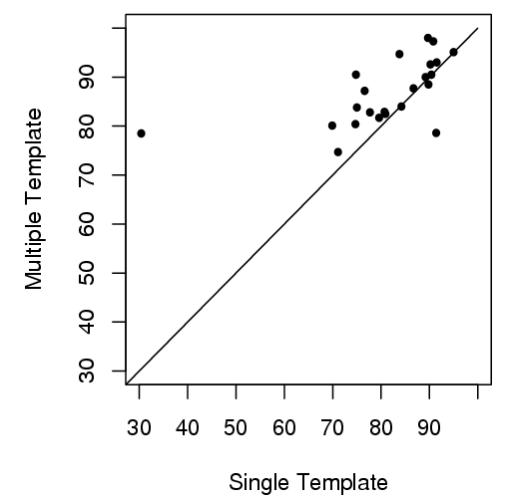
**GDT-TS scores of 23 high-accuracy targets (multi-template versus single-template)**. For 20 out of 23 domains (dots above the line), the multi-template approach yields higher GDT-TS scores than the single-template approach.

Table 2 reports the results of the multi- and single-template methods on the high-accuracy domains. The average GDT-TS score for the multi- and single-template approaches is 86.7 and 81.0 respectively. The average difference is 5.7. We conduct a paired t-test on the scores (t-value = 2.51, the degree of freedom = 22). The p-value of getting an average difference >= 5.7 is 0.01 under the hypothesis that there is no difference between the multi- and single-template methods. According to the standard 0.05 threshold, the difference is significant. Among 20 out of 23 high-accuracy targets, the multi-template combination method generates better models than the single-template method as shown in Figure 3.

**Table 2:** The results of the multiple- and single-template methods on 23 CASP7 high-accuracy domains.

Domain Id	Multi	Single	Multi – Single
T0288	83.8	75.0	8.8
T0290	97.3	90.8	6.5
T0291	78.6	91.4	-12.8
T0292_1	87.7	86.7	1.0
T0292_2	74.7	71.1	3.6
T0295_1	88.5	89.8	-1.3
T0295_2	90.0	89.2	0.8
T0302	80.1	69.9	10.2
T0303_1	82.8	77.7	5.1
T0305	93.0	91.5	1.5
T0308	90.5	74.8	15.7
T0313	80.4	74.7	5.7
T0315	94.7	83.8	10.9
T0317	81.7	79.6	2.1
T0324_1	87.2	76.6	10.6
T0324_2	78.5	30.4	48.1
T0332	82.9	80.7	2.2
T0339_2	84.0	84.2	-0.2
T0340	90.5	90.4	0.1
T0345	95.1	95.0	0.1
T0346	98.0	89.7	8.3
T0359	82.5	80.9	1.6
T0366	92.6	90.2	2.4

Average	86.7	81.0	5.7

In this experiment, we compare two methods on individual domains as in CASP7. The multiple-domain proteins are split into domains according to the CASP7 domain definition. Column 1 lists the target id and the domain index for multi-domain proteins. Other columns list the GDT-TS scores of two methods and their differences. For 20 out of 23 targets, the multi-template combination algorithm yields higher GDT-TS score than the single-template method.

### Comparison with the Best Template Approach on the CASP7 Comparative Modeling Targets

A very challenging problem of comparative modeling is to improve the model accuracy over the best templates [14,52,53]. The series of the community-wide experiments from CASP1 to CASP6 show that few methods can consistently improve the model accuracy over the best templates or even the top ranked templates. A couple of recent methods [29,54,55] and the latest CASP7 experiment show that in some cases comparative modeling and refinement can improve model quality over the best templates. Thus, here we test if our multiple template combination algorithm can improve structure modeling over the best templates on the comparative modeling targets.

We use the best templates for the comparative modeling targets provided at the CASP7 web site. We select 27 targets whose best templates can be identified by PSI-BLAST to compare the multi- and best template methods. We also use the PSI-BLAST alignment between the best templates and the targets to generate structure models.

**Figure 4:**
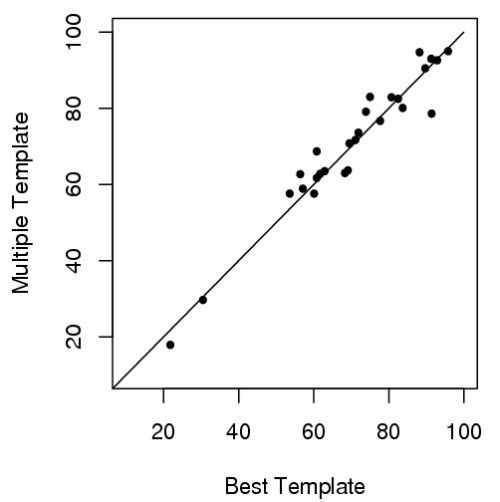
**GDT-TS scores of the 27 comparative modeling targets (multi-template versus best-template)**. For 16 out of 27 targets (points above the line), the multi-template approach yields higher GDT-TS scores than the best-template approach.

As shown in Table 3 and Figure 4, among 27 targets, the multi-template combination method produces better models for 16 targets, worse models for 10 targets, and the same quality model for 1 target. Thus, the multi-template algorithm produces better models for 62% of the targets. However, on average, the GDT-TS score is only slightly increased by .5.

**Table 3:** The GDT-TS scores of the multi- and best template methods on the 27 CASP7 comparative modeling targets.

Target Id	Best Template	Best	Multi	Multi – Best
T0291	1JPAA	91.4	78.6	-12.8
T0293	1NV9A	33.7	32.6	-1.0
T0295	1ZQ9A	75.0	83.0	8.0
T0297	1BWPA	61.7	62.8	1.1
T0298	1MB4A	69.5	70.8	1.3
T0302	1AGRE	83.7	80.1	-3.6
T0303	1GO7A	60.8	68.7	7.9
T0305	1FH7A	91.3	93.0	1.7
T0315	1J6OA	88.2	94.7	6.5
T0316	1VL2A	21.8	17.9	-3.9
T0318	1LAMA	57.1	58.9	1.8
T0323	1MPGA	53.6	57.6	4.0
T0324	2AH5A	73.9	79.1	5.2
T0329	2AH5A	68.3	63.0	-5.3
T0330	2AH5A	56.4	62.7	6.3
T0332	1ZJRA	80.7	82.9	2.2
T0339	1P3WB	77.7	76.7	-1.0
T0340	1G9OA	89.7	90.5	0.8
T0345	1GP1A	95.8	95.0	-0.8
T0359	2BYGA	82.5	82.5	0.0
T0362	2GF6A	71.9	73.6	1.7
T0364	2AV9B	71.1	71.7	0.6
T0366	2FNEB	92.9	92.6	-0.3
T0371	1YDFA	60.8	61.7	0.9
T0379	2B0CA	62.9	63.5	0.6
T0380	2FHQA	69.1	63.7	-5.4
T0381	1MKMA	60.1	57.6	-2.5

Average		70.43	70.94	0.51

Column 1 lists target id and column 2 the best template id (PDB code + chain id). The chain id of the single-chain template protein is always "A". Other columns list the GDT-TS scores of the two methods and their differences, respectively.

We conduct a pairwise t-test on the GDT-TS scores of two approaches (t-value = 0.6, degree of freedom = 26). The p-value of the statistical analysis is 0.28. Thus, on average, the GDT-TS score increase of using multiple templates over the best possible template is not significant.

### Comparison with the other Servers and Human Predictors on the CASP7 High-Accuracy Modeling Targets

We compare the accuracy of our multi-template combination algorithm against the other servers that participated in the CASP7 community-wide experiment. Two of our servers, FOLDpro and 3Dpro, along with 65 other servers were evaluated in the category of high-accuracy structure prediction in CASP7. FOLDpro and 3Dpro used the same multi-template combination algorithm, but run on different versions of non-redundant sequence databases. Table 4 reports the official total GDT-TS scores of the first models of top 20 (out of 67) servers on the 28 high-accuracy domains in CASP7. The GDT-TS scores are directly taken from the official CASP7 evaluation. The data is kindly provided by Dr. Yang Zhang at his web site [56]. The results show that our methods FOLDpro and 3Dpro using multiple-template combination algorithm were ranked second and third respectively. The performance of our methods that use the simple combination of PSI-BLAST alignments is very close to the best method (Zhang-Server) that extracts distance restraints from multiple templates used in conjunction with a more sophisticated and complicated model generation tool TASSER.

**Table 4:** The total GDT-TS scores of the top 20 out of 67 servers on the 28 high-accuracy comparative modeling domains in CASP7.

Predictors	Rank	GDT-TS	ZScore
Zhang-Server [31,91,92]	1	2415	17.7
FOLDpro [37]	2	2389	16.6
3Dpro [37]	3	2379	15.9
UNI-EID expm [93]	4	2350	13.9
CIRCLE [42]	5	2341	12.7
RAPTOR [94]	6	2328	12.6
ROBETTA [61,95,96]	7	2328	12.1
beautshotbase [97]	8	2328	11.9
FAMS [42]	9	2327	12.0
FUNCTION [42]	10	2321	11.9
HHpred1 [39]	11	2314	11.2
Pcons6 [98]	12	2309	11.0
Huber-Torda-Srv [99]	13	2306	10.8
RAPTOR-ACE [100]	14	2300	10.7
SP3 [63]	15	2295	10.4
HHpred2 [39]	16	2294	10.6
SPARKS2 [101]	17	2293	10.2
HHpred3 [39]	18	2291	10.3
beautshot [97]	19	2288	10.9
SP4 [63]	20	2287	9.8

Column 1 lists the predictor names, column 2 the ranks, column 3 the total GDT-TS scores and column 4 Z-Scores. For a model of each target, Z-score is the normalized GDT-TS score: (*x *- *u*)/*σ *calculated as in [52,90]. Here, *x *is the GDT-TS score of the model; *u *is the average GDT-TS score of all predicted models for the target; *σ *is the standard deviation. For each predictors, the Z-scores for all targets are summed into a total Z-Score to compare them as shown in the table.

Furthermore, using the more strict measure GDT-HA specially designed for high-accuracy models, FOLDpro and 3Dpro are ranked third and fifth according to the official CASP assessment [57,58]. We also compare the performance of automated servers with the human predictors. The comparision is not fair because human predictions started from the server predictions and took much longer time (about three weeks of human versus two days of server). However, it is still interesting to see what values human predictions can add on the high accuracy targets.

Table 5 reports the top 10 predictors among the 116 human and 67 server predictors in the high-accuracy structure modeling in CASP7. The data is kindly provided by Dr. Yang Zhang at his website [59]. The results show that three automated servers Zhang-Server, FOLDpro, and 3Dpro yielded the performance comparable to the best human predictors that used much longer time and took a pool of server predictions as inputs. Our servers FOLDpro and 3Dpro were ranked fifth and sixth, respectively. Zhang-Server is better than all human predictors except for Zhang human predictor from the same group. FOLDpro is better than 113 out of 116 human predictors.

**Table 5:** The total GDT-TS scores of the top 10 out of 183 predictors (67 servers + 116 human predictors) in the category of the high-accuracy structure modeling in CASP7.

Predictors	Rank	GDT-TS	Z-Score
Zhang	1	2425	17.7
Zhang-Server*	2	2415	17.0
fams-ace	3	2396	15.9
TASSER	4	2390	15.9
FOLDpro*	5	2389	15.9
3Dpro*	6	2379	15.4
fams-multi	7	2378	15.0
CIRCLE-FAMS	8	2368	14.1
hPredGrp	9	2368	14.1
CHIMERA	10	2361	13.5

* denotes server predictors. Z-score is defined in the caption of Table 4.

Since the main goal of this paper is to demonstrate the effectiveness of using multiple templates instead of evaluating different predictors in CASP7, readers are advised to check out the CASP7 assessment papers published in the Proteins journal for the thorough evaluations using different measures such as GDT-TS, GDT-HA and AL0.

### Good and Bad Examples of Using Multiple Templates

The correct usage of multiple homologous templates in general but, not always can improve comparative modeling [60,61]. As the reviewer point out, the effectiveness of multi-template modeling may correlate with the number of templates, the structural consistency amongst templates, and query-template similarity. Clarifying their relation can help decide when to use multiple templates. However, currently no quantitative measure of the relationship can be derived. Thus, here we discuss a few good and bad examples to illustrate the advantages and disadvantages of using multiple templates.

**Figure 5:**
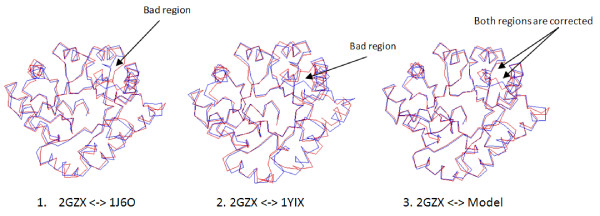
**An good example (CASP7 target T0315) of using multiple templates to improve model quality**. (1) The superimposition of the experimental structure (PDB code: 2GZX) and the best template (PDB code: 1J6O). Blue and red lines represent the backbone of the experimental and template structures, respectively. One bad region is identified. (2) The superimposition of the experimental structure and a good template (PDB code: 1YIX). One bad region is identified. (3) The superimposition of the experimental structure and the model generated by 3Dpro during CASP7, based on multiple templates). Two bad regions in (1) and (2) are corrected in the model (3). Most other regions of the model are also closer to the experimental structure than the two templates.

Figure 5 shows a good example (T0315, length = 257). The best template for the target is protein 1J6O in the PDB, whose Root Mean Square Distance (RMSD) with the experimental structure (2GCX) is 1.33 Å for 240-residue long aligned region. The other good template is 1YIX having RMSD 1.44 Å for 244-residue long aligned region. However, both templates have some bad regions that do not align well with the experimental structure. Figure 5(1) and Figure 5(2) show two different bad loop regions for the two templates, respectively. Interestingly, the two bad regions are corrected in the model generated by using multiple templates as in Figure 5(3). In addition to the obvious improvement in the two regions, the backbone of most other regions of the model are also closer to the experimental structure than the two templates. The RMSD between the model and the experimental structure is 0.88 Å for 248-residue long aligned regions. This example clearly shows that combining the complementary good templates can improve model quality.

The other good example is a two-domain target T0324, where the improvement of using multiple templates on the second domain (4-helix bundle) is drastic. The GDT-TS score of the second domain is increased by 48 (Table 1). A close examination reveals that the top template does not provide the right orientations for the helices, which are corrected by the other templates.

However, multi-template combination may occasionally decrease the model quality when there is a very good template that is much closer to the target than all other templates. One such an example is T0291 (length = 310). The most significant and best template is 1JPA, whose RMSD with the experimental structure (2GSF) is 0.72 Å for 264-residue long aligned region. The sequence identity and PSI-BLAST e-value is 81% and 10^-153^. The RMSD between other three significant templates (2SRC, 1Y57, 1KSW) and the experimental structure is 3.22 Å for 250-residue aligned region, 2.33 Å for 262-residue aligned region, and 3.20 Å for 250-residue aligned region respectively. These three templates are much more different from the target structure than the best template. However, because the alignments between these three templates and the experimental structure are very significant (e-value < 10^-142 ^and sequence identity > 40%), these three templates together with the best one are combined to generate models for the target.

The RMSD between the model and the experimental structure is 2.63 Å for 271-residue long alignment, which is better than two templates (2SRC and 1KSW), but worse than the best template (1JPA). This example shows that combining multiple templates may not help if one template is much closer to the true structure than all other templates.

### Why does the Multi-Template Approach Work in General?

We consider the following factors contributing to the improvement. First, statistically, the average model generated from multiple templates is better than the single top template on average. This is due to the ability of Modeller extracting spatial restraints from multiple templates and weighting them effectively. The weighting scheme can weight the most likely distance restraints more, resulting in picking correct aligned regions from different templates in most cases [8]. The effective combination of good aligned regions of different templates can improve comparative modeling, particulary in the cases where multiple templates provide the complementary good coverage of the target.

Second, multiple templates contain sequence and structure conservation and variation information (e.g. conserved distance restraints), which is not available in a single template. The evolutionary information is often useful to improve both secondary and tertiary structure prediction [58,61-63]

Third, PSI-BLAST can generate good local alignments for homologous proteins (comparative modeling), particulary for close homologs (easy comparative modeling). In fact, PSI-BLAST, a profile-sequence local alignment method, generates better alignments for the comparative modeling (or easy) targets than the profile-profile alignment methods (e.g., ClustalW [64], T-Coffee [65], COACH [66], and Palign [67]) we tested, which is consistent with the previous observations (Dr. Kimmen Sjölander, personal communication). However, profile-profile alignment methods are shown to produce better alignments on hard targets (<= 20% identity) [39] and to have stronger fold recognition power than profile-sequence alignment methods [37,39,66-74].

Fourth, the ranking of homologous templates by PSI-BLAST e-values for a target protein is also reasonably good, although not perfect. The greedy combination of PSI-BLAST templates and alignments into a multiple alignment centered on the target protein is effective for comparative modeling as shown in our experiments.

## Conclusion

In this study, we have developed a novel and effective algorithm of selecting and combining multiple templates and their alignments generated by PSI-BLAST, which significantly improves the quality of comparative modeling over the traditional single-template approach on the CASP7 benchmark. The alignment files of both the single-template approach and the multi-template approach are available [see Additional files 1 and 2]. The comparative modeling web server of using multiple templates is accessible at the MULTICOM website [75].

## Methods

### Multi-Template Combination Algorithm

We develop a novel and effective multi-template combination algorithm to select and combine template-target alignments for comparative modeling (Figure 1). The algorithm uses as inputs the template proteins identified by the PDB-BLAST approach [50,68], similar as ISS [76] and DOUBLE-BLAST approaches [77].

The PDB-BLAST approach searches for structure templates for a target protein in two steps. First, it uses PSI-BLAST [50] to search the target protein against the NCBI non-redundant sequence database [78] to build a profile. The e-value threshold (-h option) for building iterative profiles is set to 10^-10^; the number of iterations (-j option) is set to 3; and the e-value threshold (-e option) for inclusion in the final profile is set to 0.001. Second, it uses PSI-BLAST to search the profile against a template library compiled from the proteins in the Protein Data Bank [49] to identify structure templates homologous to the target protein. The number of iterations in this step is set to 5 and all other parameters to the default values. We select the template-target alignments with e-value < 0.001 for the combination.

Each returned template protein may have one or more local alignments with the target protein. Each alignment is associated with an e-value that measures its similarity significance. We use the logarithm of the e-value as the significance score. The smaller the score, the more significant is the alignment.

**Figure 6:**
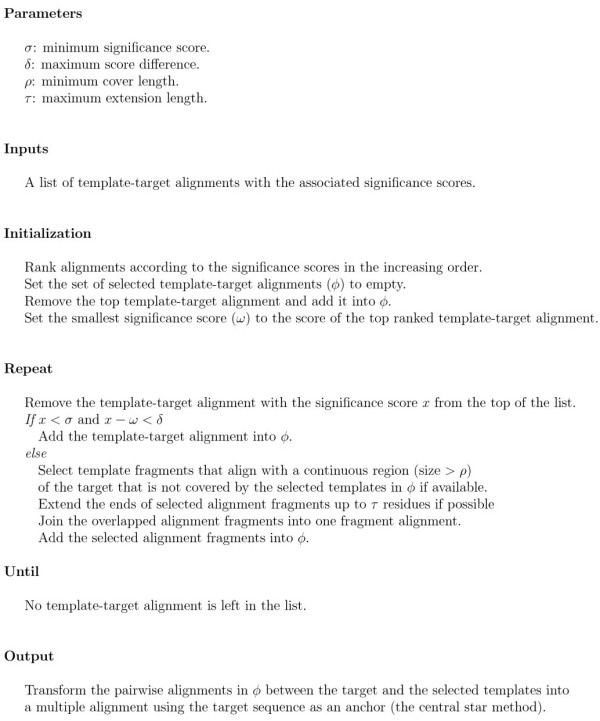
Multi-Template Selection and Combination Algorithm.

The only inputs to the algorithm are the template-target alignments and the associated significance scores. Thus, with some modification, the algorithm can be applied to other template-based structure prediction methods with different scoring schemes (e.g. z-score in threading). Figure 6 briefly describes the algorithm. The algorithm always uses the most significant template-target alignment. The other significant alignments whose significance score is less than *σ *and close to the score of the most significant template-target alignment within the threshold *δ *are also automatically included. The less significant template-target alignments are used only when they can align with a continuous region of the target whose size is bigger than *ρ *and which is not covered by the previously selected template-target alignments. And only the alignment fragments that align with the uncovered regions are excised and included. *ρ *controls the size of the selected fragments, which can be used to avoid selecting very small fragments.

One template-target alignment may provide several fragments that align with disjoint, uncovered regions of the target. The alignment fragments can be extended at both ends up to *τ *residues if possible, which may create a linker to overlap with the other fragments from the same template or the alignments from other templates. After the extension, the overlapped or non-gapped alignment fragments excised from the same template-target alignment are combined into one alignment. The join of fragments can introduce long-range constraints, which is useful for structure modeling.

The alignments between the target and the selected templates are combined into a multiple alignment using the target sequence as an anchor, the same as the central star multiple alignment approach [79,80] employed by the construction of position specific scoring matrix (PSSM) [81,82] in PSI-BLAST [50]. The multiple alignment together with the template structures are fed into comparative modeling tools such as Modeller to generate structure models for the target protein. Modeller weights the spatial restraints extracted from multiple templates according to sequence identity appropriately [8], resulting in generating a better average model than a single template in most situations.

The algorithm is fully parametrized (Figure 6). All parameters can be tuned. In our experiments, we set *σ *to -20, *δ *to 12, *ρ *to 5, and *τ *to 5. The parameters were tuned on the CASP6 targets and blindly tested on the CASP7 targets. The most important parameters are *σ *and *δ *because the combination of the whole significant templates contributes most to the improvement of structure modeling. *ρ *and *τ *usually play a less important role because short un-covered regions of a target protein are usually well handled by the loop building module of Modeller.

### Limitation and Future Work

The multi-template combination algorithm developed here is very effective for comparative modeling where target and template proteins have strong homologous relationship and their alignments are rather accurate. But the method does not always produce good results for hard template-based structure prediction, i.e. protein fold recognition.

We had applied the similar algorithm to the hard fold recognition targets during CASP7 using the global alignments generated by COACH [66] (results not shown here). The algorithm works well when the structure templates and their alignments with the target are consistent, but performs poorly when structure templates or sequence-structure alignments have a lot of spatial inconsistency (particularly in unalignable regions). The models generated from multiple inconsistent target-template alignments usually contain a lot of atom-atom clashes – a quantitative indicator of spatial inconsistency. One possible reason is that the current version of Modeller cannot well resolve a large amount of conflicting distance restraints within multiple templates as also observed in [60].

Thus, although multiple templates are useful in general, a more sophisticated way of combining them and removing inconsistency is required to achieve better performance for fold recognition and threading, where the template-target relatedness and alignment are uncertain and less reliable than comparative modeling. The spatial inconsistency may be removed either in model reconstruction phase as in TASSER or in alignment optimization phase as in [83].

One possible direction is to use structure alignment tools such as DALI [84], SSAP [85], VAST [86], CE [87], and TM-align [88] to check the structure consistency between templates and to remove inconsistent templates and alignments (work in progress).

## Authors' contributions

JC designed the algorithm, wrote the program, carried out the experiments, and authored the manuscript. All authors read and approved the final manuscript.

## Supplementary Material

Additional file 1 **The description of supplemental materials**. This file describes how to use supplemental materials. It is a text file that can be viewed by any text viewer. Click here for file

Additional file 2 **The compressed file of supplemental materials**. The file can be decompressed by winzip on Windows or by tar xzf on Linux. The file includes query-template alignments of both the single-template approach and the multi-template approach that were used to generate the structure models for the CASP7 comparative modeling targets. Click here for file

## References

[B1] Vitkup D, Melamud E, Moult J, Sander C (2001). Completeness in structural genomics. Nature Struct Biol.

[B2] Brenner S (2001). A tour of structural genomics. Nature Rev Genet.

[B3] Westbrook J, Feng Z, Chen L, Yang H, Berman H (2003). The protein data bank and structural geomics. Nucleic Acids Res.

[B4] Browne W, North A, Philips D, Brew K, Vanaman T, Hill R (1969). A possible three-dimensional structure of bovine alpha-lactalbumin based on that of hen.s egg-white lysozyme. J Mol Biol.

[B5] Blundell T, Sibanda B, Sternberg M, Thornton J (1987). Knowledge-based prediction of protein structures and the design of novel molecules. Nature.

[B6] Greer J (1990). Comparative modeling methods: Application to the family of the mammalian serine proteases. Proteins.

[B7] Levitt M (1992). Accurate modeling of protein conformation by automatic segment matching. J Mol Biol.

[B8] Sali A, Blundell T (1993). Comparative protein modelling by satisfaction of spatial restraints. J Mol Biol.

[B9] Koehl P, Delarue M (1994). Application of a self-consistent mean field theory to predict protein side-chains conformation and estimate their conformational entropy. J Mol Biol.

[B10] Sali A (1998). 100,000 protein structures for the biologist. Nat Struct Biol.

[B11] Marti-Renom M, Stuart A, Fiser A, Sanchez R, Melo F, Sali A (2000). Comparative protein structure modeling of genes and genomes. Annu Rev Biophys Biomol Struct.

[B12] Sali A (2001). Target practice. Nat Struct Biol.

[B13] Bates P, Kelley L, MacCallum R, Sternberg M (2001). Enhancement of protein modeling by human intervention in applying the automatic programs 3D-JIGSAW and 3D-PSSM. Proteins.

[B14] Tramontano A, Leplae R, Morea V (2001). Analysis and assessment of comparative modeling in CASP4. Proteins.

[B15] Kolinski A, Betancourt M, Kihara D, Rotkiewicz P, Skolnick J (2001). Generalized comparative modeling (GENECOMP): a combination of sequence comparison, threading, and lattice modeling for protein structure prediction and refinement. Proteins.

[B16] Lambert C, Leonard N, Bolle X, Depiereux E (2002). ESyPred3D: Prediction of proteins 3D structures. Bioinformatics.

[B17] Petrey D, Xiang Z, Tang C, Xie L, Gimpelev M, Mitros T, Soto C, Goldsmith-Fischman S, Kernytsky A, Schlessinger A (2003). Using multiple structure alignments, fast model building, and energetic analysis in fold recognition and homology modeling. Proteins.

[B18] Schwede T, Kopp J, Guex N, Peitsch M (2004). SWISS-MODEL: An automated protein homology-modeling server. Nucleic Acids Res.

[B19] Tramontano A (2006). Protein Struture Prediction: Concepts and Applications.

[B20] Sanchez R, Sali A (1997). Advances in comparative protein-structure modeling. Curr Opin Struct Biol.

[B21] Petrey D, Honig B (2005). Protein structure prediction: inroads to biology. Mol Cell.

[B22] Venclovas C (2003). Comparative modeling in CASP5: progress is evident, but alignment errors remain a significant hindrance. Proteins.

[B23] Venclovas C, Margelevicius M (2005). Comparative modeling in CASP6 using consensus approach to template selection, sequence-structure alignment, and structure assessment. Proteins.

[B24] Blundell T, Sternberg M (1985). Computer-aided design in protein engineering. Trends Biotechnol.

[B25] Sutcliffe M, Haneef I, Carney D, Blundell T (1987). Knowledge based modelling of homologous proteins, Part I: Three-dimensional frameworks derived from the simultaneous superposition of multiple structures. Protein Eng.

[B26] Blundell T, Barlow D, Sibanda B, Thornton J, Taylor W, Tickle I, Sternberg M, Pitts J, Haneef I, Hemmings A (1986). Three-Dimensional structural aspects of the design of new protein molecules. Phil Trans Roy Soc Lond Ser A.

[B27] Overington J, Johnson M, Sali A, Blundell T (1990). Tertiary structural constraints on protein evolutionary diversity; templates, key residues and structure prediction. Proc Roy Soc Lond sect B.

[B28] Al-Lazikani B, Sheinerman F, Honig B (1998). Combining multiple structure and sequence alignments to improve sequence detection and alignment: application to SH2 domains of Janus kinase. PNAS.

[B29] Zhang Y, Skolnick J (2004). Automated Structure Prediction of Weakly Homologous Proteins on a Genomic Scale. PNAS.

[B30] Skolnick J, Kihara D, Zhang Y (2004). Development and large scale bechmark testing of the PROSPECTOR 3.0 threading algorithm. Proteins.

[B31] Zhang Y, Arakaki A, Skolnick J (2005). TASSER: an automated method for the prediction of protein tertiary structure in CASP6. Proteins.

[B32] Moult J, Hubbard T, Bryant SH, Fidelis K, Pedersen JT (1997). Critical assessment of methods of protein structure prediction (CASP): round II. Proteins Suppl.

[B33] Moult J, Hubbard T, Bryant SH, Fidelis K, Pedersen JT (1999). Critical assessment of methods of protein structure prediction (CASP): round III. Proteins Suppl.

[B34] Moult J, Fidelis K, Zemla A, Hubbard T (2001). Critical Assessment of Methods of Protein Structure Prediction (CASP): Round IV. Proteins.

[B35] Moult J, Fidelis K, Zemla A, Hubbard T (2003). Critical assessment of methods of protein structure prediction (CASP)-round V. Proteins.

[B36] Moult J, Fidelis K, Tramontano A, Rost B, Hubbard T (2005). Critical assessment of methods of protein structure prediction (CASP) – round VI. Proteins.

[B37] Cheng J, Baldi P (2006). A Machine Learning Information Retrieval Approach to Protein Fold Recognition. Bioinformatics.

[B38] Cheng J (2006). Machine Learning Algorithms for Protein Structure Prediction.

[B39] Söding J (2005). Protein homology detection by HMM-HMM comparison. Bioinformatics.

[B40] Pandit S, Zhang Y, Skolnick J (2006). TASSER-Lite: an automated tool for protein comparative modeling. Biophys J.

[B41] Zhou H, Pandit S, Lee S, Borreguero J, Chen H, Wroblewska L, Skolnick J (2007). Analysis of TASSER-based CASP7 protein structure prediction results. Proteins.

[B42] Ogata K, Umeyama H (2000). An automatic homology modeling method consisting of database searches and simulated annealing. J Mol Graphics Mod.

[B43] Sali A, Potterton L, Yuan F, van Vlijmen H, Karplus M (1995). Evaluation of comparative protein modeling by MODELLER. Proteins.

[B44] Fiser A, Do R, Sali A (2000). Modeling of loops in protein structures. Protein Science.

[B45] Levitt M (1992). Accurate modeling of protein conformation by automatic segment matching. J Mol Biol.

[B46] Guex N, Peitsch M (1997). SWISS-MODEL and Swiss-PdbViewer: an environment for comparative protein modeling. Electrophoresis.

[B47] Schwede T, Diemand A, Guex N, Peitsch M (2000). Protein structure computing in the geomic era. Res Microbiol.

[B48] Zemla A (2003). LGA: a method for finding 3D similarities in protein structures. Nucleic Acids Research.

[B49] Berman H, Westbrook J, Feng Z, Gilliland G, Bhat T, Weissig H, Shindyalov I, Bourne P (2000). The Protein Data Bank. Nucl Acids Res.

[B50] Altschul S, Madden T, Schaffer A, Zhang J, Zhang Z, Miller W, Lipman D (1997). Gapped BLAST and PSI-BLAST: a new generation of protein database search programs. Nucleic Acids Research.

[B51] Tress M, andO Grana IE, Lopez G, Valencia A (2005). Assessment of predictions submitted for the CASP6 comparative modeling category. Proteins.

[B52] Tramontano A, Morea V (2003). Assessment of homology based prediction in CASP5. Proteins.

[B53] Wallner B, Elofsson A (2005). All are not equal. A benchmark of different homology modeling programs. Protein Science.

[B54] Zhang Y, Skolnick J (2005). The protein structure prediction problem could be solved using the current PDB library. P N A S.

[B55] Misura K, Chivian D, Rohl C, Kim D, Baker D (2006). Physically realistic homology models built with ROSETTA can be more accurate than their templates. Proc Natl Acad Sci USA.

[B56] CASP7 high accuracy GDT-TS results (server). http://zhang.bioinformatics.ku.edu/casp7/22.html.

[B57] Battey J, Kopp J, Bordoli L, Read R, Clarke N, Schwede T (2007). Automated server predictions in CASP7. Proteins.

[B58] Read R, Chavali G (2007). Assessment of CASP7 predictions in the high accuracy template-based modeling category. Proteins.

[B59] CASP7 high accuracy GDT-TS results (human and server). http://zhang.bioinformatics.ku.edu/casp7/32.html.

[B60] Wu S, Zhang Y (2007). LOMETS: a local meta-threading-server for protein structure prediction. Nucleic Acids Research.

[B61] Das R, Qian B, Raman S, Vernon R, Thompson J, Bradley P, Khare S, Tyka M, Bhat D, Chivian D, Kim D, Sheffler W, Malmstrom L, Wollacot A, Wang C, Andre I, Baker D (2007). Structure prediction for CASP7 targets using extensive all-atom refinement with Rosetta@home. Proteins.

[B62] Rost B, Sander C (1994). Combining evolutionary information and neural networks to predict protein secondary structure. Proteins.

[B63] Zhou H, Zhou Y (2005). Fold recognition by combining sequence profiles derived from evolution and from depth-dependent structural alignment of fragments. Proteins.

[B64] Thompson J, Higgins D, Gibson T (1994). CLUSTALW: improving the sensitivity of progressive multiple sequence alignment through sequence weighting, position-specific gap penalties and weight matrix choice. Nucleic Acids Res.

[B65] Notredame C, Higgins D, Heringa J (2000). T-Coffee: A novel method for multiple sequence alignment. J Mol Biol.

[B66] Edgar R, Sjölander K (2004). COACH: profile-profile alignment of protein families using hidden Markov models. Bioinformatics.

[B67] Ohlson T, Wallner B, Elofsson A (2004). Profile-profile methods provide improved fold-recognition. A study of different profile-profile alignment methods. Proteins.

[B68] Rychlewski L, Jaroszewski L, Li W, Godzik A (2000). Comparison of sequence profiles. Strategies for structural predictions using sequence information. Protein Sci.

[B69] Yona G, Levitt M (2002). Within teh twilight zone: a sensitive profile-profile comparison tool based on information theory. J Mol Biol.

[B70] Mitelman D, Sadreyev R, Grishin N (2003). Probabilistic scoring measures for profile-profile comparison yield more accurate short seed alignments. Bioinformatics.

[B71] Ginalski K, Pas J, Wyrwicz L, vonGrotthuss M, Bujnicki J, Rychlewski L (2003). ORFeus: Detection of distant homology using sequence profiles and predicted secondary structure. Nucleic Acids Res.

[B72] Sadreyev R, Grishin N (2003). COMPASS: A tool for comparison of multiple protein alignments with assessment of statistical significance. J Mol Biol.

[B73] Wallner B, Fang H, Ohlson T, Frey-Skott J, Elofsson A (2004). Using evolutionary information for the query and target improves fold recognition. Proteins.

[B74] Marti-Renom M, Madhusudhan M, Sali A (2004). Alignment of protein sequences by their profiles. Protein Sci.

[B75] MULTICOM. http://casp.rnet.missouri.edu/multicom/multicom.html.

[B76] Park J, Teichmann S, Hubbard T, Chothia C (1997). Intermediate sequences increase the detection of homology between sequences. J Mol Biol.

[B77] Karplus K, Barrett C, Hughey R (1998). Hidden Markov models for detecting remote protein homologies. Bioinformatics.

[B78] Pruitt K, Tatusova T, Maglott D (2003). NCBI Reference sequence project: update and current status. Nucleic Acids Res.

[B79] Gusfield D (1993). Efficient methods for multiple sequence alignment with guaranteed error bounds. Bulletin of Mathematical Biology.

[B80] Gusfield D (1997). Algorithms on Strings, Trees and Sequences: Computer science and computational biology.

[B81] Gribskov M, McLachlan M, Eisenberg D (1987). Profile analysis: detection of distantly related proteins. PNAS.

[B82] Henikoff S, Wallace J, Brown J (1990). Finding protein similarity with nucleotide sequence database. Methods in Enzymology.

[B83] Joo K, Lee J, Lee S, Seo J, Lee S, Lee J (2007). High accuracy template based modeling by global optimization. Proteins.

[B84] Holm L, Sander C (1993). Protein Structure Comparison by Alignment of Distance Matrices. J Mol Biol.

[B85] Taylor W, Flores T, Orengo C (1994). Multiple protein structure alignment. Protein Sci.

[B86] Gibrat JF, Madej T, Bryant SH (1996). Surprising similarities in structure comparison. Curr Opin Struct Biol.

[B87] Shindyalov IN, Bourne PE (1998). Protein Structure Alignment by incremental combinatorial extension (CE) of the optimal path. Protein Engineering.

[B88] Zhang Y, Skolnick J (2005). TM-align: a protein structure alignment algorithm based on the TM-score. Nucleic Acids Research.

[B89] ForCASP. http://www.forcasp.org.

[B90] Tress M, Ezkurdia I, Grana O, Lopez G, Valencia A (2005). Assessment of predictions submitted for the CASP6 comparative modeling category. Proteins.

[B91] Wu S, Skolnick J, Zhang Y (2007). Ab initio modeling of small proteins by iterative TASSER simulations. BMC Biology.

[B92] Zhang Y (2007). Template-based modeling and free modeling by I-TASSER in CASP7. Proteins.

[B93] Debe D, Danzer J, Goddard W, Poleksic A (2006). STRUCTFAST: Protein sequence remote homology detection and alignment using novel dynamic programming and profile-profile scoring. Proteins.

[B94] Xu J, Li M, Kim D, Xu Y (2003). RAPTOR: Optimal Protein Threading by Linear Programming. J Bioinformatics and Computational Biology.

[B95] Kim D, Chivian D, Baker D (2004). Protein structure prediction and analysis using the Robetta server. Nuclear Acids Research.

[B96] Chivian D, Kim D, Malmstrom L, Bradley P, Robertson T, Murphy P, Strauss C, Bonneau R, Rohl C, Baker D (2003). Automated prediction of CASP-5 structures using the Robetta server. Proteins.

[B97] Fischer D (2003). 3DS3 and 3DS5 3D-SHOTGUN Meta-Predictors in CAFASP3. Proteins.

[B98] Wallner B, Elofsson A (2005). Pcons5: combining consensus, structural evaluation and fold recognition scores. Bioinformatics.

[B99] Torda A, Procter J, Huber T (2004). Wurst: a protein threading server with a structural scoring function, sequence profiles and optimised substitution matrices. Nucl Acids Res.

[B100] Xu J, Yu L, Li M (2005). Consensus fold recognition by predicted model quality. Asian-Pacific Bioinformatics Conference (APBC).

[B101] Zhou H, Zhou Y (2004). Quantifying the effect of burial of amino acid residues on protein stability. Proteins.

